# Epithelial-Mesenchymal Plasticity Induced by Discontinuous Exposure to TGFβ1 Promotes Tumour Growth

**DOI:** 10.3390/biology11071046

**Published:** 2022-07-12

**Authors:** Mafalda Santos, Marta Ferreira, Patrícia Oliveira, Nuno Mendes, Ana André, André F. Vieira, Joana B. Nunes, Joana Carvalho, Sara Rocha, Mafalda Azevedo, Daniel Ferreira, Inês Reis, João Vinagre, Joana Paredes, Alireza Heravi-Moussavi, Jorge Lima, Valdemar Máximo, Angela Burleigh, Calvin Roskelley, Fátima Carneiro, David Huntsman, Carla Oliveira

**Affiliations:** 1 I3S, Instituto de Investigação e Inovação em Saúde, 4200-135 Porto, Portugal; mafaldas@ipatimup.pt (M.S.); martaf@ipatimup.pt (M.F.); poliveira@ipatimup.pt (P.O.); nmendes@ipatimup.pt (N.M.); aandre@ipatimup.pt (A.A.); avieira@ipatimup.pt (A.F.V.); j.c.bras_gomes_nunes@lumc.nl (J.B.N.); jcarvalho@ipatimup.pt (J.C.); srocha@ipatimup.pt (S.R.); daniel.ferreira@i3s.up.pt (D.F.); jvinagre@ipatimup.pt (J.V.); jparedes@ipatimup.pt (J.P.); jlima@ipatimup.pt (J.L.); vmaximo@ipatimup.pt (V.M.); fcarneiro@ipatimup.pt (F.C.); 2 Ipatimup, Instituto de Patologia e Imunologia Molecular da Universidade do Porto, 4200-465 Porto, Portugal; azevedom@mskcc.org (M.A.); inescaladoreis@chporto.min-saude.pt (I.R.); 3 INEB, Instituto de Engenharia Biomédica—Rua Alfredo Allen, 4200-135 Porto, Portugal; 4 Department of Pathology and Oncology, Faculty of Medicine, University of Porto, 4200-319 Porto, Portugal; 5 British Columbia Cancer Agency, Vancouver, BC V5Z4E6, Canada; amoussavi@bcgsc.ca (A.H.-M.); aburleigh@alumni.ubc.ca (A.B.); calvin.roskelley@ubc.ca (C.R.); dhuntsma@bccancer.bc.ca (D.H.)

**Keywords:** MET, cellular heterogeneity, self-Renewal, EMT, tumourigenic potential

## Abstract

**Simple Summary:**

In this manuscript, we used a non-genetically manipulated EMT/MET cell line model to demonstrate that epithelial mesenchymal plasticity occurring in normal cells generates co-existing phenotypically and functionally divergent cell subpopulations which result in fast growing tumours in vivo.

**Abstract:**

Transitions between epithelial and mesenchymal cellular states (EMT/MET) contribute to cancer progression. We hypothesize that EMT followed by MET promotes cell population heterogeneity, favouring tumour growth. We developed an EMT model by on and off exposure of epithelial EpH4 cells (E-cells) to TGFβ1 that mimics phenotypic EMT (M-cells) and MET. We aimed at understanding whether phenotypic MET is accompanied by molecular and functional reversion back to epithelia by using RNA sequencing, immunofluorescence (IF), proliferation, wound healing, focus formation and mamosphere formation assays as well as cell xenografts in nude mice. Phenotypic reverted epithelial cells (RE-cells) obtained after MET induction presented epithelial morphologies and proliferation rates resembling E cells. However, the RE transcriptomic profile and IF staining of epithelial and mesenchymal markers revealed a uniquely heterogeneous mixture of cell subpopulations with a high self-renewal ability. RE cell heterogeneity was stably maintained for long periods after TGFβ1 removal both in vitro and in large tumours derived from the nude mice. Overall, we show that phenotypic reverted epithelial cells (RE cells) do not return to the molecular and functional epithelial state and present mesenchymal features related to aggressiveness and cellular heterogeneity that favour tumour growth in vivo. This work strengthens epithelial cell reprogramming and cellular heterogeneity fostered by inflammatory cues as a tumour growth-promoting factor in vivo.

## 1. Introduction

Epithelial-to-mesenchymal transition (EMT) and the reverse process, mesenchymal-to-epithelial transition (MET), are biological mechanisms naturally occurring during embryogenesis and regeneration [1,2]. Although contradictory at first, the role of EMT and MET in cancer progression and metastasis has now been fully acknowledged [2,3,4,5,6]. While EMT enables epithelial cancer cell dissemination, bestowing cells with increased invasion, migration and stem cell properties, MET facilitates the establishment of these cells at secondary sites [3,7,8]. EMT and MET were previously seen as strict transition states where cells acquire specific phenotypes and molecular signatures. However, this biological programme is very dynamic and cannot be accurately defined by limited sets of markers or phenotypic changes. The concomitant expression of epithelial and mesenchymal markers in cancer cells suggests the occurrence of hybrid EMT states [1,8,9,10]. This cellular plasticity confers advantageous features to cancer cells, conferring them with increased adaptability to microenvironment cues and resistance to several stressors [11]. Supporting this, Armstrong et al. showed that >75% of circulating tumour cells (CTCs), isolated from patients with metastatic prostate and breast cancers, exhibited intermediate phenotype and stem cell markers [12]. Moreover, Yu et al. observed that CTCs from breast cancer patients show a varying proportion of epithelial and mesenchymal markers associated with different breast cancer subtypes and treatment responses [13].

Many strategies have been described to induce EMT in vitro, such as artificially induced overexpression of transcription factors, namely Snail and Twist1 [14,15,16], or treatment with growth factors or cytokines such as TGFβ1, EGF and NGF [1,17,18]. These in vitro as well as in vivo studies have strengthened the hypothesis that EMT followed by MET occurs at different levels of cancer progression. Hugo et al. showed that the primary tumours derived from breast cancer cells exhibited evidence of EMT at the invasive front, while the derived metastasis expressed high levels of E-cadherin, suggesting MET [8]. Tsai et al. showed that after activation of the EMT inducer Twist1, cancer cells disseminated into the blood circulation, but Twist1 was inactivated to induce MET, allowing disseminated cancer cells to metastasize [19]. In line with this, Ocaña et al. demonstrated that loss of the EMT inducer Prrx1, together with the acquisition of an epithelial phenotype and stem cell properties, were required for cancer cells to form metastases in vivo, reinforcing MET as an important event for cancer colonization [20].

Several reports also correlate EMT drivers with increased stemness and the prevalence of tumour-initiating cells (TICs) [21,22]. Available EMT/MET models study mammary stem cells and cancer stem cells separately, or they rely on cell transformation through activation of an oncogene, such as KRAS [22]. The EMT driver TGFβ was able to trigger increased breast TICs in claudin-low breast cancer cell lines [23]. However, in a different study, it was suggested that TGFβ had an inhibitory role in breast TICs [24]. The molecular contexts in which these events occurred were not disclosed. Increased stemness causes phenotypic diversity in cancer cells [25]. Dynamic and reversible cell state transitions cause phenotypic variability and allow for quick adaptive responses to a variety of stressful situations, which can have a significant impact on cancer progression [26]. Herein, we hypothesize that EMT followed by MET promotes cell population heterogeneity, and this favours tumour growth. We characterized and explored an EMT/MET model and unveiled that MET generates population heterogeneity, which may drive tumour growth in vivo.

## 2. Materials and Methods

### 2.1. Cell Culture

EpH4 [25], provided by Dr. Angela Burleigh and Dr. Calvin Roskelley, was cultured as in [26]. The MCF10A cell line was cultured in DMEM/F12 Glutamax™ medium supplemented with horse serum (5%, Lonza, Basel, Switzerland), recombinant human insulin (5 μg/mL), penicillin-streptomycin (1%, Invitrogen), hydrocortisone (500 ng/mL, Sigma-Aldrich, Burlington, VT, USA), cholera toxin (20 ng/mL, Sigma-Aldrich, Burlington, VT, USA) and recombinant human epidermal growth factor (20 ng/mL, Sigma-Aldrich, Burlington, VT, USA). Cell authentication was performed by Ipatimup’s Cell Lines Bank using Powerplex16 STR amplification (Promega, Madison, WI, USA). The M cells were obtained as in [26] using Transforming Growth Factor-β1 (TGFβ1, Sigma-Aldrich, Burlington, VT, USA). The RE cells were obtained as in [26] (Figure S1).

### 2.2. RNA Extraction and Quantification

RNA extraction, cDNA conversion and quantitative-Real-Time-PCR (qRT-PCR) from the E, M and RE cells were performed as in [26]. The qRT-PCR assays used were TaqMan Gene Expression (ThermoFisher, Waltham, MA, USA) and PrimeTime-qPCR (IDT, San Diego, CA, USA): CDH1 (Mm00486909_g1), Ocln (Mm.PT.47.16166845), Mgat3 (Mm00483213_m1), Zeb2 (Mm.PT.47.13169136), Vim (Mm01333430_m1), CDH2 (Mm.PT.45.14052292), Twist1 (Mm00492575_m1), GAPDH (Mm99999915_g1) and 18S (Hs99999901_s1). The data were analysed by the 2^(−ΔΔCT)^ method [27] and compared using the Mann–Whitney test [28].

### 2.3. Immunocytochemistry

The E, M and RE cells were fixed with methanol (Merck, Burlington, VT, USA), blocked using 3%BSA-PBS-0.5%Tween20 (Sigma-Aldrich, Burlington, VT, USA) and incubated with anti-Snail (L70G2, 1:50, Cell Signaling, Danvers, TX, USA), anti-MMP2 (42-5D11, 1:50, Calbiochem, San Diego, CA, USA) and anti-mouse Alexa 488 (A-11029, 1:500, ThermoFisher, Waltham, MA, USA). For E-cadherin and Fibronectin co-immunocytochemistry, the cells were fixed and blocked as previously described and co-incubated with anti-E-cadherin (1:50, Cell Signaling, Danvers, TX, USA), anti-Fibronectin (1:50, Santa Cruz, Dallas, TX, USA) overnight at 4 °C and anti-rabbit or anti-mouse Alexa 488 or 594, respectively, at room temperature for 1 h (1:500, ThermoFisher, Waltham, MA, USA). Coverslips were mounted using Vectashield-DAPI mounting medium (Vector Laboratories, Burlingam, CA, USA). Images were taken with ZeissImager.Z1AxioCamMRm (Zeiss, Oberkochen, Germany). These experiments were repeated at least three times, where several fields were photographed to represent the maximum intensity projection.

### 2.4. Whole-Transcriptome Sequencing

The RNA for whole-transcriptome sequencing (RNAseq) was extracted from 2 biological replicates of EpH-4 cell line stages (E, M and RE) using the mirVana miRNA Isolation Kit (Invitrogen, OR, USA) according to the kit’s instruction manual and maintained at −80 °C until further processing. Genomic DNA was removed using RNase-Free-DNase (Qiagen, Hilden, Germany) and purified using an RNEasy Mini Kit (Qiagen, Hilden, Germany). The RNA quality was analysed using an Agilent2100 Bioanalyzer (RIN > 9.7). The E, M and RE cells were sequenced using an Illumina Genome Analyzer (n = 2) as a service at BCCA. The unique reads were mapped to the NCBI-m37 mouse genome using Bowtie, TopHat2 and differentially expressed genes (DEGs) detected using the edgeR R package. Genes with a log2fold change >1 or <−1 and corrected *p* < 1.00 × 10^−2^ were considered DEGs (Figure S3). Statistics performed using the R. ClusterProfiler R package (version 4.0.5) was used for the assessment of significantly enriched GO terms and pathways (padj < 5.00 × 10^−2^). The hierarchical clustering was performed using the heatmap.2 function from the gplots R package, with the complete method used as an argument for the hclust function that computed the hierarchical clustering. We used the Euclidean method to compute the distance and the complete method to perform the hierarchical clustering. The whole-transcriptome sequencing data were deposited at GEO, and its accession number was GSE181008.

### 2.5. BrdU Assay

The E, M and RE cells were incubated with 1 μL of BrdU solution (ThermoFisher, Waltham, MA, USA) for 90 min, washed with a phosphate-buffered saline solution (PBS) and fixed using 4% formaldehyde (Sigma-Aldrich, Burlington, VT, USA). Next, the cells were treated with 2M-HCl (Merck, Burlington, VT, USA), washed with PBS-0, 5% Tween 20*–*0 and 5% BSA (Sigma-Aldrich, Burlington, VT, USA) and incubated with anti-BrdU antibody (1:10, ThermoFisher, Waltham, MA, USA) and anti-mouse Ig-FITC (1:100, ThermoFisher, Waltham, MA, USA). The coverslips were mounted using Vectashield-DAPI mounting medium (Vector Laboratories, Burlingam, CA, USA). Images were taken with ZeissImager.Z1AxioCamMRm (Zeiss, Oberkochen, Germany), and the stained nuclei were counted. Statistical analysis was performed using the Mann–Whitney test [28].

### 2.6. Focus Formation Assay

The E, M and RE cells were plated in 100-mm plates and grown for 21 days (TGFβ1-supplemented for M-cells). Brightfield images were taken for phenotype comparison [27].

### 2.7. Wound Healing Assay

Wounds were produced in confluent E, M and RE cell cultures using a filter tip. Brightfield images of several E, M and RE cell wounds were taken at distinct timepoints (maximum: 12 h) [28].

### 2.8. In Vivo Tumourigenesis Assay, H&E Staining and KI67 Immunohistochemistry

First, 1 × 106/2 × 106 E, M or RE cells resuspended in 100 μL of DMEM-F12 were inoculated in the mammary fat pad of 5–6-week-old female NIH(S)II-nu/nu mice. For the pilot study, 2 mice were inoculated with E, M or RE cells in the mammary fat pad (total n = 6). For the in vivo tumourigenicity assay, 5 mice with double E/RE cell inoculation and 5 mice with single M cell inoculation (total n = 15) were used. Two mice were excluded due to unrelated health issues. For the in vivo syngeneic transplantation assay, 1-mm^3^ sections of 1 E, 1 M and 3 RE tumours were transplanted into the mammary fat pads of 5 mice. The tumours were measured with callipers, and the volumes were estimated using (Width ∗ Length^2^)/2. The experiments were carried out in accordance with European Guidelines for the Care and Use of Laboratory Animals, Directive-2010/63/UE and national regulations (Diário da República-1.^a^ série-N.°151). All mice were humanely euthanized. All tumours were formalin-fixed, paraffin-embedded and stained for hematoxylin and eosin. The immunohistochemistry were performed for Ki67 as in [29] (ThermoFisher, Waltham, MA, USA). Three representative fields of each tumour were selected, and the Ki67-stained nuclei were counted using D-sight software (A.Menarini Diagnostics, Florence, Italy). The sections of 2 E tumours, 3 M tumours and 6 RE tumours were conserved in RNA (ThermoFisher, Waltham, MA, USA) and later used for RNA extraction, cDNA conversion and qRT-PCR as in [26]. Statistical analysis was performed using the Mann–Whitney test [28].

### 2.9. First Passage Mammosphere Formation Assay

The E, M and RE cells were plated (750 cells/cm^2^) in 6 1.2% polyhema-coated wells (Sigma-Aldrich, Burlington, VT, USA). The mammospheres were grown in DMEM/F12 medium supplemented with 5 mg/mL insulin, 0.5 mg/mL hydrocortisone, 2% B27 and 20 ng/mL epidermal growth factor [29]. The mammospheres were counted after 5 days. Statistical analysis was performed using the Mann–Whitney test [28].

## 3. Results

### 3.1. Phenotypic EMT/MET vs. Molecular EMT/MET

**Figure 1:**
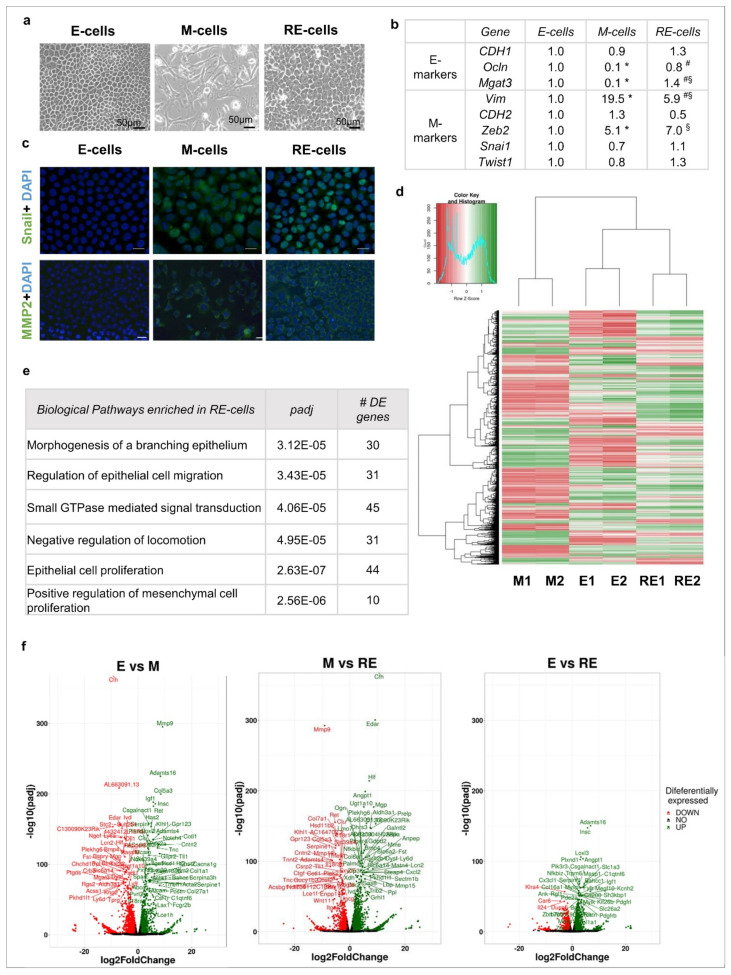
The RE cells underwent MET and displayed a transcriptomic signature that partially resembled E cells. (**a**) Brightfield images of E, M and RE cells. (**b**) RNA expression analysis of EMT-associated markers in E, M and RE-cells (qRT-PCR, where data were analysed using the Mann–Whitney test; * = *p* < 5.00 × 10^−2^ for E vs. M cells, § = *p* < 5.00 × 10^−2^ for E vs. RE cells and # = *p* < 5.00 × 10^−2^ for M vs. RE cells). (**c**) Immunofluorescence staining for Snail, MMP2 and DAPI as indicated. RE cells showed strong staining for both Snail and MMP2, indicating that they retained M features. (**d**) Heat map of differentially expressed genes assessed by RNAseq (0.6 > fold change > 1.5, *p* < 1.00 × 10^−2^). Z-scored values expressed from −1 to 1 (from red to green, representing low to high expression levels, respectively). M1 and M2 = M cell reps 1 and 2, E1 and E2 = E cell reps 1 and 2 and RE1 and RE2 = RE cell reps 1 and 2. (**e**) Significantly enriched biological pathways derived from the 1288 differentially expressed genes specific to RE cells. (**f**) Volcano plots highlighting the differentially expressed genes in all the comparisons among the different cell lines.

To decipher the mechanisms underlying naturally occurring MET, we established and characterized an in vitro EMT/MET model using the near-normal EpH4 mouse mammary epithelial cell line (E cells) exposed to the EMT inducer TGFβ1 [30]. This non-cancer cell line was selected to prevent cancer-related bias. Moreover, this model has a homogenous nature both in terms of brightfield morphology and epithelial and mesenchymal marker expression [30]. After 7 days of TGFβ1 treatment, the E cells acquired a fibroblastoid phenotype, resembling mesenchymal cells (M cells, Figure 1a). The TGFβ1 was then removed from the culture medium, and after another 4 days, brightfield microscopy revealed widespread recovery of an epithelial phenotype (reverted epithelial (RE) cells, Figure 1a and Figure S1). To confirm EMT induction and reversion, the E, M and RE cells were characterized for expression of the epithelial (*CDH1*, *Ocln* and *Mgat3*) and mesenchymal (*Vim*, *CDH2*, *Zeb2*, *Snai1* and *Twist1*) markers by qRT-PCR (Figure 1b). As we previously reported, we did not observe a significant alteration in *CDH1* expression, but the function of the corresponding protein E-cadherin was impaired due to downregulation of the *Mgat3* expression in the M cells, which is responsible for GnT-III-mediated glycosylation [30]. Moreover, the M cells displayed significant downregulation of other epithelial markers (*Ocln*) and upregulation of certain mesenchymal markers (*Vim* and *Zeb2*). In the RE cells, the expression of *Ocln*, *Mgat3* and *Vim* returned to levels similar to those of the E cells. In contrast, *Zeb2* expression remained elevated in the RE cells, while *Snai1* and *Twist1* exhibited no alterations across the E, M and RE cells (Figure 1b). Immunofluorescence staining of these E, M and RE cells for the *Snail* and *MMP2* mesenchymal markers showed the classical EMT pattern, since they are not expressed in E cells and are expressed in M cells, but they remained unchanged in the RE cells compared with the M cells, suggesting that the RE cells did not fully revert to the epithelial state (Figure 1c). Overall, the phenotypic changes, gene expression and immunofluorescence results support that the current EMT model mimics phenotypic EMT (M-cells) and MET but also suggests that phenotypic MET may not be accompanied by molecular and functional reversion back to epithelia.

### 3.2. Phenotypic MET Is Not Supported by Complete Molecular Reversion Back to Epithelia

We next used whole-transcriptome sequencing (RNAseq) to explore the differences and similarities between the E, M and RE cells. A good correlation was observed between the expression pattern obtained through qRT-PCR and RNAseq for the epithelial and mesenchymal markers (Figure S2). This validation allowed the use of RNAseq data to assess the expression variation of other EMT-associated markers that supported EMT and partial MET (S2Fig). The RNAseq data were also used to identify differentially expressed genes (DEGs) across the transcriptomic landscapes of the E, M and RE cells by comparing (1) the E and M cells, (2) M and RE cells and (3) E and RE cells (fold change >1.50 or <0.66, *p* < 1.00 × 10^−2^, Figure S3). These DEGs were then submitted for double hierarchical clustering analysis (Figure 1d). Although the RE cell signature was more closely related to the E cells than the M cells overall, this cell state presented its own transcriptomic landscape. Indeed, the expression of 1288 genes was modulated only in the RE cells while remaining stable during EMT (both in the E and M cells). Among the top-ranking biological pathways, there were “morphogenesis of a branching epithelium”, “regulation of epithelial cell migration”, “small GTPase-mediated signal transduction” and also “negative regulation of locomotion”. Both “epithelial cell proliferation” and “positive regulation of mesenchymal cell proliferation” were also part of the highly significant biological pathways in RE cells (Figure 1e). These data further support that MET-generated epithelial-looking cells differ from their original epithelial counterparts at the molecular level, but they also differ significantly from the M-state cells from which they arose (Figure 1d–f and Figure S4). A deeper analysis of the biological functions and pathways significantly enriched across the experiment (Figure S5) showed that “Cellular Growth and Proliferation”, “Migration”, “Stemness” and “Cancer” were affected in these transitions. These observations further support the molecular differences between E, M and RE cells, highlighting that even though our in vitro model was developed using a near-normal cell line, a significant association with aggressiveness and cancer-related features was detected upon EMT/MET induction.

### 3.3. Phenotypic MET Generates E-like, M-like and Novel Cellular Subpopulations

**Figure 2:**
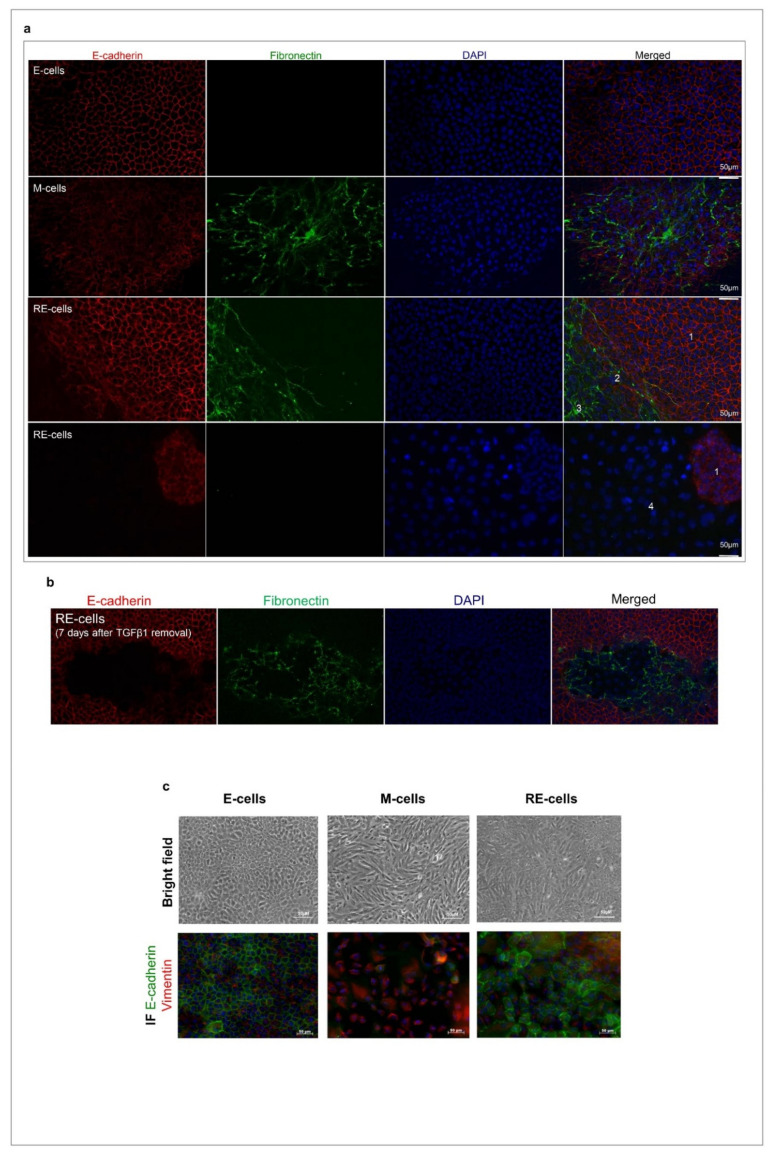
RE cells are a mixture of different cellular subpopulations. (**a**) Immunofluorescence staining for E-cadherin (red), Fibronectin (green) and DAPI (blue) of E, M and RE cells. Four different subpopulations were identified: 1: Ecad+/Fn−; 2: Ecad+/Fn+; 3: Ecad−/Fn+ and 4: Ecad−/Fn−).(**b**) RE cells retain their E-cadherin or Fibronectin staining heterogeneity even if reversion time is longer than 4 days. Immunofluorescence images are for RE cells grown for 7 days after TGFβ1 removal from the culture medium. (**c**) Brightfield images of MCF10A E, M and RE cells (upper panel) and immunofluorescence staining for E-cadherin (green) and Vimentin (red) of MCF10A E, M and RE cells (bottom panel).

Given that RE cells seem to be transcriptionally heterogeneous and have a set of quite specific molecular features, we next assessed the immunoexpression of the epithelial marker E-cadherin and the mesenchymal marker Fibronectin in situ in E, M and RE cells (Figure 2a). The E cells displayed homogenous E-cadherin membrane staining and lacked Fibronectin, while the M cells showed an irregular staining of membranous E-cadherin (described in [30]) and high expression of extracellular Fibronectin (Figure 2a). The RE cells revealed a far more complex expression pattern of these two markers that evidenced the existence of four distinct subpopulations (Ecad+/Fn+, Ecad+/Fn−, Ecad−/Fn+ and Ecad−/Fn−), some of which were previously absent from the E and M cells (Figure 2a). The most striking of all RE cell populations were those lacking E-cadherin expression (Ecad−/Fn+ and Ecad−/Fn−), which appeared exclusively in the RE cells, a molecular change that is generally associated with EMT. To better assess the extent of an RE cell’s phenotypic heterogeneity, full slides of stained RE cells were scanned. Of notice, no field was homogeneous for any of the four RE subpopulations previously described, reinforcing their spatial co-existence (Figure S6). To understand whether this heterogeneity was temporary and part of the reversal process back to the E state, the RE cells were cultured for longer periods without TGFβ1. After 7 days, the same four subpopulations were still observed in the RE cell cultures (Figure 2b). We could confirm this was not a specificity of the EpH4 on/off model, as the same transdifferentiation protocol applied to the human immortalized normal breast epithelial cell line MCF10A cells returned similar results. Brightfield images of the MCF-10A E, M and RE cells showed that the RE cells were a mixture of E-like and M-like cells (Figure 2c, upper panel). We could not optimize the Fibronectin staining in this cell line, so we chose Vimentin as a mesenchymal marker. Unlike EpH4 M cells, MCF10A M cells completely lose E-cadherin expression (Figure 2c, bottom panel). On the other hand, MCF10-A cells behave similarly to EpH4 cells after TGFβ removal from the media regarding heterogeneous E-cadherin and Vimentin staining across the culture, confirming that upon TGFβ on/off exposure, some cell populations do not fully revert to the E state.

### 3.4. Cellular Heterogeneity, Generated after Phenotypic MET In Vitro, Creates Functional Heterogeneity

**Figure 3:**
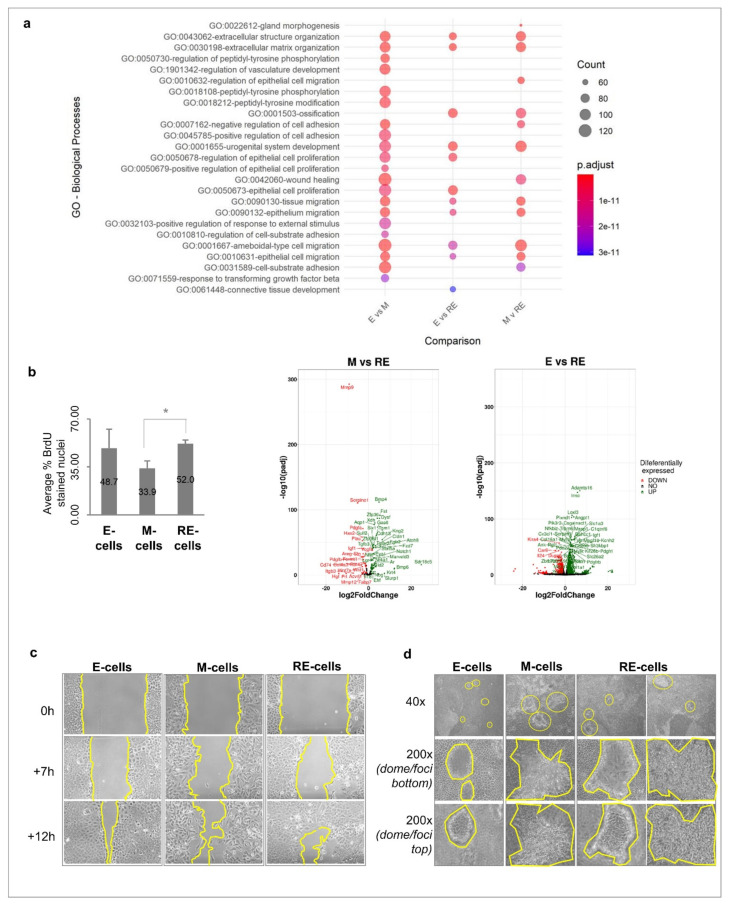
RE cells exhibit a high proliferation rate, heterogeneous migration pattern and focus formation assay. (**a**) Biological processes deregulated across comparisons (padj < 0.05), displaying that RE cells have a unique biological identity different from both E and M cells. (**b**) Cell proliferation analysis in terms of percentage of BrdU stained nuclei (per total number of DAPI-stained nuclei, n = 3, where * = *p* < 0.05 for MvsRE comparison). Volcano plots show upregulation of proliferation-related genes in RE cells when compared to M and E cells. (**c**) RE cells exhibit mixed cell migration patterns, resembling both E and M cells. Wound healing brightfield images of E, M and RE cells were taken at different timepoints (0, 7 and 12 h, 100×). (**d**) Brightfield images of E, M and RE cells grown for 21 days in plastic and normal culture medium. Top panel shows general view of the 21-day cultured E, M and RE cells (40×). Middle panel shows bottom layer of non-transformed cells surrounding dome-like structures or foci (200×). Bottom panel shows top layer of dome-like structures or foci obtained after 21 days of culture of E, M and RE cells (200×).

Given that RE cells represent a heterogeneous cellular population which is stable for several days in culture, we explored RE cells’ functional behaviour in comparison to E and M cells. Moreover, we wanted to test the hints of aggressiveness observed in the transcriptomics analysis which were eventually triggered by these transitions. For that, we analysed the cell proliferation, cell behaviour when growing into a wound and growth pattern in a focus formation assay, as several related biological functions were found to be enriched in RE cells in the transcriptomics analysis (Figure 3a and Figure S5). BrdU incorporation revealed that the E and RE cells displayed a higher proliferation rate than the M cells, but only the RE cells were statistically different from the M cells (49% and 52% for E and RE cells, respectively, vs. 34% for M cells, *p* < 0.05, Figure 3b). In fact, when comparing the RE cells with either the M or E cells for proliferation-related DEGs, the M cells presented a larger number of downregulated proliferation-associated genes than the E cells when both were compared with the RE cells, likely explaining why only the M cells differed from the RE cells in the vitro experiment (Figure 3a and Figure S5). The wound healing assays photographed and analysed at several timepoints showed that the M cells in the wound were mainly isolated, while a sheet of seemingly epithelial cells covered the wound area in the E cells (Figure 3c). In the RE cells, we observed both isolated cells resembling those seen in the M cells as well as areas with high cellular density, resembling the epithelial sheets seen in the E cells. In summary, the RE cells proliferated similarly to the E cells and faster than the M cells while displaying both isolated cells and epithelial sheets covering the wound, reminiscent of both the E and M cells (Figure 3c). To understand whether the RE cells acquired aggressive cancer-like features (Figure S5), we next ran a focus formation assay. For this, E, M and RE cells were cultured for 21 days, and their morphological differences were evaluated by brightfield microscopy (Figure 3d). The E cells generated a few small and spherical structures with defined edges which, according to Gordon et al., could be considered dome-like structures resembling non-malignant mammary glands [31]. The M cells displayed a high number of foci with large and irregular edges but not dome-like structures, which is an indicator of increased aggressiveness. The RE cells displayed both E-like domes and M-like foci. Together with the previous data, this supports RE cells as an entity with unique and heterogeneous phenotypes, retaining both E-like and M-like features in the population. These results supported the hypothesis that MET may confer a more aggressive phenotype to otherwise immortalized normal cells.

### 3.5. Cellular Heterogeneity, Generated after Phenotypic MET In Vitro, Is Maintained in Tumours Growing In Vivo

**Figure 4:**
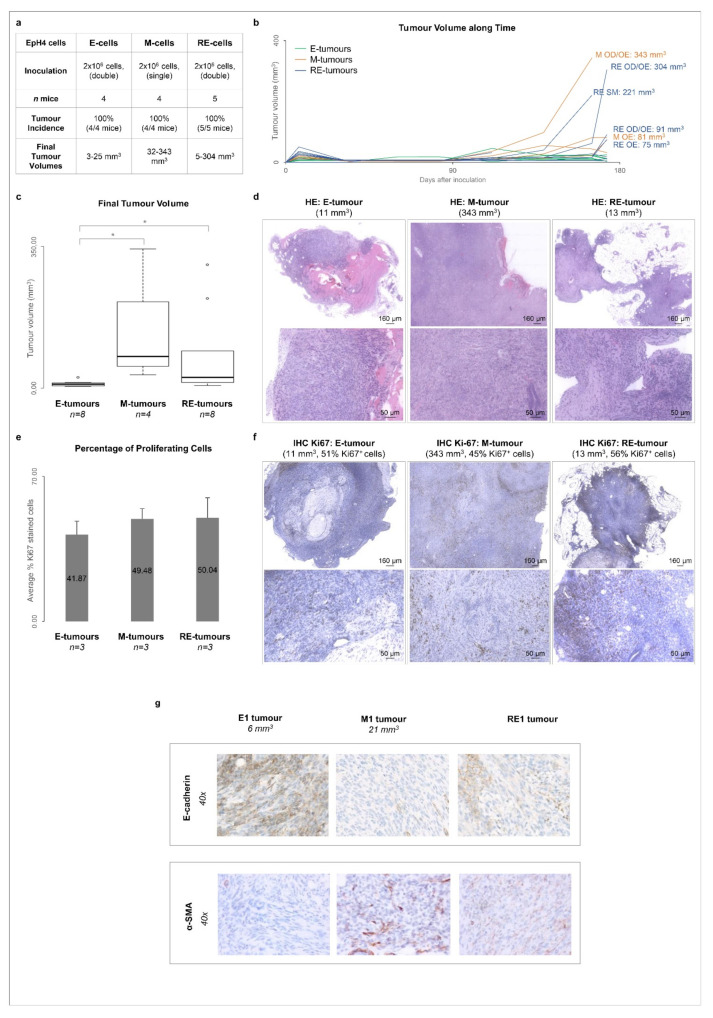
M and RE cells displayed higher in vivo tumourigenicity than E cells without retaining the original in vitro RNA profile. (**a**) Summary of the in vivo tumourigenicity assay performed with E, M and RE cells. (**b**) Growth curves representing the tumour volumes over time (E tumours in green, M tumours in orange and RE tumours in blue). (**c**) Final tumour volumes obtained for each cell type (* for *p* < 5.00 × 10^−2^, with data analysis performed with the Mann–Whitney test). (**d**) Representative images of hematoxylin and eosin staining of E, M and RE tumours. Top and bottom images show different magnifications. (**e**) Average percentage of cells positive for Ki67 staining in 3 E, 3 M and 3 RE tumours. (**f**) Representative images of immunohistochemistry staining for Ki67. Top and bottom images show different magnifications. (**g**) E-cadherin and α-SMA immunohistochemistry staining of representative E, M and RE tumours.

Our RNAseq data suggest that RE cells are enriched in deregulated cancer-related pathways when compared with their E and M counterparts (Figure S5). Therefore, we performed an in vivo pilot study where cells that underwent EMT and MET were inoculated in the mammary fat pads of athymic nude mice. It is noteworthy that EpH4 cells have been described as non-tumourigenic [32]. In a pilot study, one out of two (1/2) mice inoculated with E cells developed a tumour (6 mm^3^ at day 145), and one out of two (1/2) mice inoculated with M cells developed another tumour (21 mm^3^ at day 132) (Figure S7a). Both mice inoculated with RE cells developed larger tumours (121 and 63 mm^3^ at day 145, Figure S4a). This pilot study prompted us to assess the tumourigenicity of E, M or RE cells in a larger group of mice (n = 5, Figure 4a). In this second study, all cell types inoculated formed tumours, albeit with significantly distinct volumes. By the end of the experiment, the E tumours were significantly smaller in size (<30 mm^3^) than the M tumours (32–343 mm^3^) and RE tumours (5–304 mm^3^, Figure 4b,c) (*p* < 0.05). The M and RE tumours were similar in size. All tumours were classified as malignant sarcomatoid carcinomas upon histopathological evaluation (Figure 4d). All tumours presented evidence of hyalinization, while only the M and RE tumours displayed necrotic areas. The M tumours revealed signs of inflammation and local epidermis invasion, while the RE tumours displayed an increased cellular density (Figure 4d). The presence of mitotic nuclei and the different tumour volumes observed led us to assess the proliferation in E, M and RE-tumours by Ki67 immunostaining, but no significant differences were observed (Figure 4e,f and Figure S7b–h). The molecular differences of the E, M and RE tumours were assessed through immunohistochemistry against E-cadherin and α-SMA. The E tumours expressed E-cadherin but not α-SMA, whereas the M tumours lost E-cadherin expression and presented α-SMA staining. The RE tumours, however, expressed both markers, suggesting that the cellular heterogeneity created in vitro after MET could be maintained after long periods in vivo.

### 3.6. RE Cells Mimic M Cells in Self-Renewal Capacity In Vitro and Fast Growth of Tumour Transplants In Vivo

**Figure 5:**
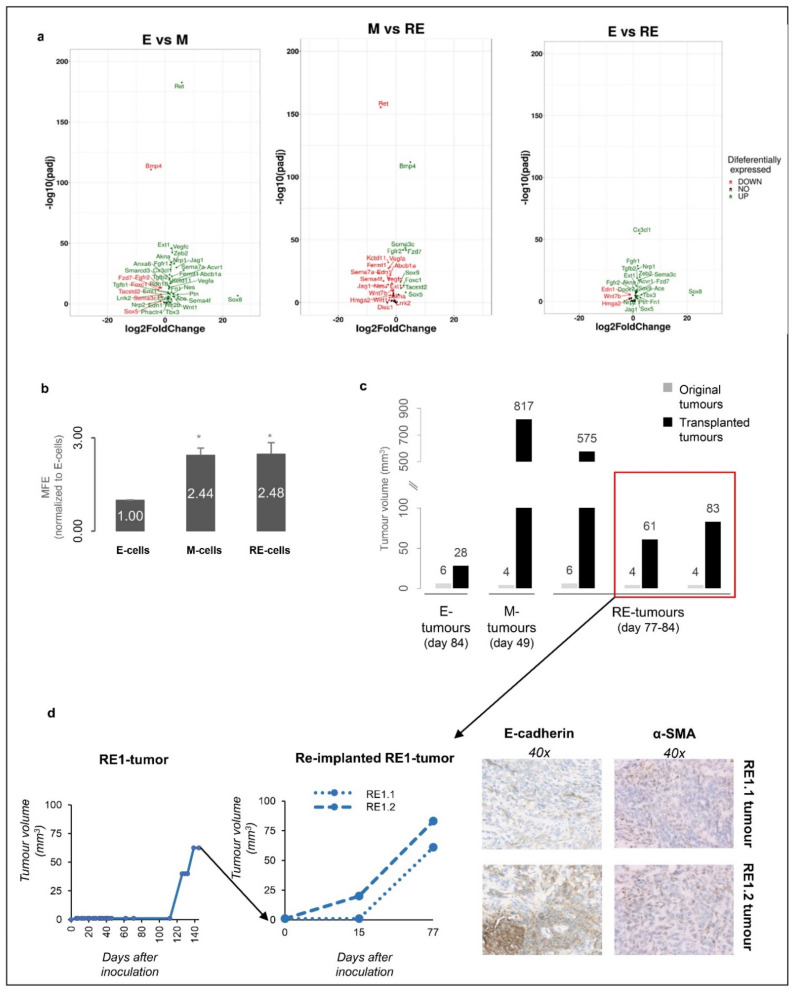
RE cells displayed increased stemness potential. (**a**) Volcano plots of stemness-related DEGs. (**b**) E, M and RE cells’ first-passage mammosphere formation efficiencies (* for *p* < 5.00 × 10^−2^). (**c**) Pilot in vivo syngeneic transplantation assay for E, M and RE tumours. Comparison shown between the final tumour volumes for the original E, M and RE tumours used (grey bars) and the corresponding tumours obtained after syngeneic transplantation (black bars) at the same time point post-inoculation or transplantation. (**d**) (left panel) Growth curve of an RE tumour in the first passage in mice (left) and after reinoculation of two tumour pieces (right). (right panel) E-cadherin and α-SMA immunohistochemistry staining of representative RE reimplanted tumours.

Our in vitro RNAseq data also revealed that not only the M cells but also the RE cells were enriched in stem cell-related pathways compared with the E cells (Figure S5 and Figure 5a), in agreement with the literature showing that EMT generates cells with increased stemness [18]. Therefore, we explored whether the M and RE cells displayed an increased self-renewal capacity in vitro by using a first-passage mammosphere assay to evaluate whether the E, M and RE cells were able to grow in anchorage-independent conditions. Both the M and RE cells displayed an increased ability to form first-passage mammospheres in comparison with the E cells (Figure 5b). Notably, the RE cells were able to form first-passage mammospheres with the same efficiency as the M cells (Figure 5b). In parallel, we tested the self-renewal ability of the E, M and RE tumours by syngeneic transplantation of small tumour fragments [33]. The histology of the transplanted tumours mimicked that of the original tumours, but the growth rate of the transplanted tumours was higher than that of the original counterparts (180 days in the original experiment vs. 49–84 days after transplantation) (Figure 5c). In particular, both the M and RE transplanted tumours displayed a growth rate higher than the E transplanted tumour, likely due to the increased self-renewal ability [34]. In particular, two reimplanted fragments of an RE tumour started their exponential growth just 15 days post-inoculation, when the original tumour took 110 days to start growing (Figure 5d, left panel). Immunostaining of these tumours with E-cadherin and α-SMA revealed similar expression patterns to that of the original tumours, further supporting that cellular heterogeneity was stable (Figure 5d, right panel). Altogether, our results show that the RE cells exhibited a high first-passage mammosphere formation efficiency, which is consistent with the faster growth of the tumour transplants in vivo.

## 4. Discussion

In this study, we showed that phenotypic reverted epithelial cells (RE cells) did not return to the epithelial state in molecular and functional terms and presented mesenchymal features related to aggressiveness and cellular heterogeneity that favour tumour growth in vivo. We selected TGFβ1 for EMT induction, as this is a naturally abundant cytokine in tissues secreted by immune and other cells which populate the tumour microenvironment [35]. Moreover, TGFβ1 supplementation and withdrawal more closely recapitulates EMT and MET occurrence under physiological conditions than strategies involving genetic manipulation [36].

We characterized cells that underwent EMT and that presented a phenotypic reversion back to epithelia (RE cells). The RE cells revealed a distinct transcriptomic profile, though they resembled E cells in their cobblestone phenotype and proliferation rate (Figure S8). The RE cells displayed mixed E and M phenotypic features, were highly heterogeneous in vitro with regard to the immunoexpression of epithelial and mesenchymal markers and retained this heterogeneity when growing into large tumours in nude mice. These results are supported by Schmidt et al. [37], who suggested that cells undergoing MET may never return to their original epithelial state, gaining aggressive features and a distinct gene expression profile.

EMT has been associated with the increased presence of TICs, tumour progression and aggressiveness [22,38,39], and MET has mainly been associated with increased colonization capacity [8]. Thus far, neither EMT nor MET have been shown to drive tumourigenesis, and the link between EMT plasticity and tumour initiation is still poorly understood. Furthermore, most EMT and MET studies rely on external transformation factors such as TWIST or SLUG [22,32,40].

Strikingly, we could demonstrate that RE cells (but not E cells) could generate large tumours in vivo, which grew even larger and faster when transplanted into different animals. These experiments demonstrated that supplementation and withdrawal of a physiologically abundant cytokine was enough to rewire the molecular program of an apparently non-tumourigenic cell line, resulting in increased tumour growth in vivo [32]. Thus far, only one study reported that genetic manipulation of Fra-1 was able to induce EMT and the transformation of Eph4 cells, depending on the TGFβ levels [32]. Our work goes beyond that observation, providing evidence that physiological EMT plasticity, without genetic manipulation, may indeed contribute to fostering tumour growth.

Unlike the homogenous E or M cells, the RE cells presented a heterogeneous expression pattern of epithelial and mesenchymal markers (Figure S6). Together with our in vivo results, these findings recall other EMT-related studies. For example, the work by Tsuji et al. showed that co-injection of EMT and non-EMT cells originated more aggressive tumours than those obtained by injections of each cell type independently [41]. Upon inoculation, the RE cells were already a mixture of EMT (M-like) and non-EMT (E-like and novel phenotype) cells, which may, in light of Tsuji et al., contribute to the observed RE tumours’ increased growth rate. Moreover, the originally homogeneous M-cells also gave rise to high-volume M tumours, similar to the RE tumours. This suggests that the M cells underwent MET in vivo as the M tumours grew deprived of a persistent TGFβ1 stimulus, unlike the M cells grown in vitro. Altogether, and in line with Tsuji et al., it is plausible to hypothesize that the intrinsic heterogeneity of RE cells and the likely in vivo MET in the M tumours might enable cellular cooperation among distinct subpopulations, promoting tumour growth. Furthermore, the RE cells showed an increased self-renewal capacity when compared with the E cells, as shown by the increased tumour growth rate upon reimplantation of the tumour fragments and by the first-passage mammosphere forming efficiency (Figure 5).

Using this model, we demonstrated that after EMT and MET, there is a visible phenotypic reversion back to epithelia, which is not accompanied by molecular and functional reversion but rather produces a stable heterogeneous cell population with increased tumourigenic potential. We believe that EMT is crucial to prime E cells into a more plastic state, culminating, after stimulus removal, in the generation of these distinct subpopulations. Therefore, our model seems to recreate the phenotypic heterogeneity commonly observed in human cancers. A wide range of theories argue that such heterogeneity may arise from clonal evolution, cancer stem cells, microenvironment cues or reversible changes in cancer cells [34,42]. Our work strongly suggests that heterogeneity may be triggered by on/off exposure to a microenvironment cue (i.e., TGFβ1), bestowing cells with aggressive features. In cancer patients, this heterogeneity is likely maintained due to crosstalk between different cancer cell subpopulations or between cancer and non-cancer cell types. Supporting these assumptions is, for example, the direct TGFβ-signalling occurring between platelets and cancer cells, inducing EMT and favouring metastization [43], the turning on or off of demethylases by different melanoma cell clones, giving rise to a mixture of cells with different tumour growth efficiencies [44], or the co-existence of epithelial, mesenchymal and hybrid cancer cell states in lung adenocarcinoma, providing these tumours with increased survival [39]. Therefore, it is likely that the cell plasticity promoted by MET together with the increased stemness observed contributed to tumour progression and metastasis. To elucidate if RE cells could have an advantage in metastasis, we would have to perform in vivo metastasis assays and characterize the metastasis to observe which population would give that advantage.

In conclusion, our model allowed us to demonstrate that EMT plasticity generates cells with a heterogeneous and unique phenotype, which results in increased stemness and the ability to form large tumours in vivo, providing evidence that inflammatory cues can influence tumour growth kinetics through EMT/MET transdifferentiation.

## 5. Conclusions

We generated a dynamic EMT/MET model for mimicking the intermittent exposure of epithelial cells to microenvironment inflammatory cues. This model revealed that upon exposure to transdifferentiation factors, normal epithelial cells become plastic, originate different cell populations and lose the ability to return to their original state. As a corollary, we propose that MET originates a heterogeneous population of cells with unique features that contribute to the transformation of non-tumourigenic cells. Identifying the players maintaining cellular heterogeneity and conferring plasticity in models such as ours may shed light on tumour progression drivers and reveal important targets for therapeutic intervention in cancer.

## Data Availability

Whole transcriptome sequencing data were deposited at GEO (https://www.ncbi.nlm.nih.gov/geo/), and their accession number is GSE181008.

## Supplementary Materials

The following supporting information can be downloaded at https://www.mdpi.com/article/10.3390/biology11071046/s1. Figure S1: Establishment of the EMT/MET in vitro model. Figure S2: Validation of the RNAseq data with qRT PCR using distinct biological replicates of E, M and RE cells. RNAseq data were highly correlated with qRT PCR data. (a) RNA expression by RNAseq and qRT PCR of the epithelial markers CDH 1, Ocln and Mgat3 in E, M and RE cells. (b) RNA expression by RNAseq and qRT PCR of the mesenchymal/EMT markers Vim, Cdh2, Zeb2, Twist 1 and Snai1 in E, M and RE cells. (c) Differential gene expression of the epithelial marker Cldn1 and the mesenchymal markers Fn1, Mmp2 and Mmp9 in comparison to EvsM, MvsRE and EvsRE. Figure S3: Number of DEGs in each comparison. Figure S4: RE cells retain upregulation of mesenchymal genes. Volcano plots showing deregulated genes associated with the biological process “positive regulation of epithelial to mesenchymal transition” in E vs. M (left) and E vs. RE (right) comparisons. Genes represented in green are upregulated in M and RE cells in the left and right panels, respectively. Figure S5: Top significantly enriched biological functions or pathways derived from the 4211 differentially expressed genes across E, M and RE cells. Figure S6: The 4 RE cell subpopulations co-exist spatially. (a) Representative images of different microscope fields of RE cells stained for E cadherin (red) and Fibronectin (green), displaying the 4 RE cells subpopulations labelled from 1–4: (1) E-cadherin^+^/Fibronectin^−^, (2) E-cadherin^+^/Fibronectin^+^, (3) E-cadherin^−^/Fibronectin^+^ and (4) E-cadherin^−^/Fibronectin^−^. DAPI (blue) is also represented, and all channels are merged. Figure S7: Mice experiments. (a) Pilot in vivo tumourigenicity assay for E, M and RE cells, with M and RE cell-originated tumours having larger volumes than those of E cells. (b) Average percentage of cells positive for Ki 67 staining in 3 E tumours, 3 M tumours and 3 RE tumours. (c–h) Representative images of immunohistochemistry staining for Ki 67 in 2 E tumours, 2 M tumours and 2 RE tumours. Top and bottom images show different magnifications. Figure S8: Summary of the phenotypic and functional properties of E, M and RE cells. Properties analysed were brightfield morphology, proliferation, wound healing closure, first passage mammosphere-forming efficiency, focus formation ability, phenotypic heterogeneity by E-cadherin/Fibronectin immunofluorescence and in vivo tumourigenicity (final tumour volumes).
