# Supplementary material for: Epithelial-Mesenchymal Plasticity Induced by Discontinuous Exposure to TGFβ1 Promotes Tumour Growth
Source: Biology (Basel). 2022 Jul 12;11(7):1046. doi: 10.3390/biology11071046 (PMC9312510; doi:10.3390/biology11071046)
Supplement: Supplementary file 1 [file biology-11-01046-s001.zip › biology-1697036-Supplementary.pdf]

Supplementary material

Epithelial-Mesenchymal Plasticity Induced by Discontinuous Exposure to TGFβ1 Promotes Tumour Growth

Mafalda Santos, Marta Ferreira, Patrícia Oliveira, Nuno Mendes, Ana André, André F. Vieira, Joana B. Nunes, Joana Carvalho, Sara Rocha, Mafalda Azevedo, Daniel Ferreira, Inês Reis, João Vinagre, Joana Paredes, Alireza Heravi-Moussavi, Jorge Lima, Valdemar Máximo, Angela Burleigh , Calvin Roskelley, Fátima Carneiro, David Huntsman and Carla Oliveira

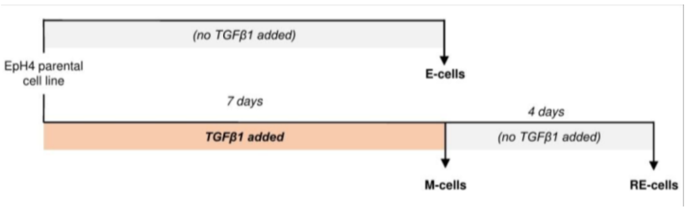

Figure S1. establishment of the EMT/MET in vitro model.

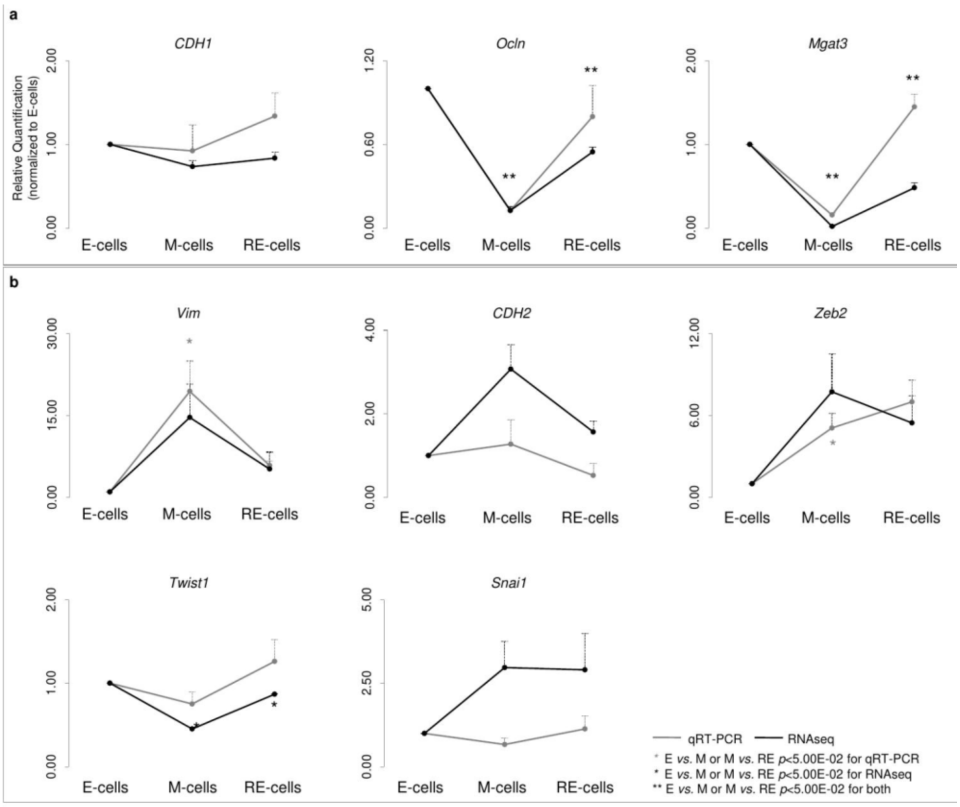

c

Gene		*Comparison*					
		**E vs M**		**M vs RE**		**E vs RE**	
		**log2(FC)**	***padj***	**log2(FC)**	***padj***	**log2(FC)**	***padj***
Epithelial Markers	*Cldn1*	-2.03	3.30E-19	3.15	9.74E-46	1.13	2.44E-06
Mesenchymal Markers	*Fn1*	2.23	2.78E-10			1.53	3.96E-05
	Mmp2			1.75	4.46E-12	2.18	4.63E-18
	Mmp9	9.17	1.02E-299	-9.13	2.46E-293		

Figure S2. Validation of the RNAseq data with qRT PCR using distinct biological replicates of E, M and RE cells. RNAseq data were highly correlated with qRT PCR data. (a) RNA expression by RNAseq and qRT PCR of the epithelial markers CDH 1, Ocln and Mgat3 in E, M and RE cells. (b) RNA expression by RNAseq and qRT PCR of the mesenchymal/EMT markers Vim, Cdh2, Zeb2, Twist 1 and Snai1 in E, M and RE cells. (c) Differential gene expression of the epithelial marker Cldn1 and the mesenchymal markers Fn1, Mmp2 and Mmp9 in comparison to EvsM, MvsRE and EvsRE.

ID	Comparison	# DEGs	Sum # DEGs
Comparison 1	E vs M	2931	7115 (4211 unique)
Comparison 2	M v RE	2336	
Comparison 3	E vs RE	1848	

Figure S3: Number of DEGs in each comparison.

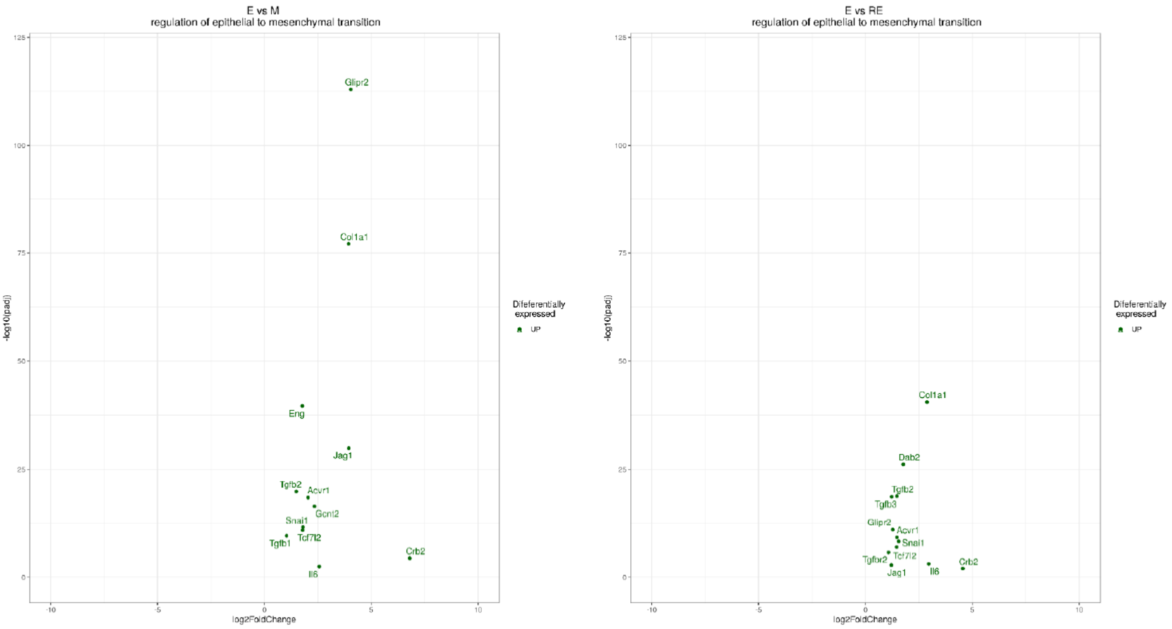

Figure S4. RE cells retain upregulation of mesenchymal genes. Volcano plots showing deregulated genes associated with the biological process “positive regulation of epithelial to mesenchymal transition” in E vs. M (left) and E vs. RE (right) comparisons. Genes represented in green are upregulated in M and RE cells in the left and right panels, respectively.

Biological Functions/Pathways	*Comparison*	*padj range*	*# DE genes*
Cellular Growth and Proliferation			173
regulation of epithelial cell proliferation	E vs RE	5.75E-13	61
regulation of epithelial cell proliferation	E vs M	5.47E-06	50
epithelial cell proliferation	E vs RE	9.20E-13	67
epithelial cell proliferation	E vs M	3.14E-06	57
epithelial cell proliferation	M v RE	5.58E-06	58
epithelial cell proliferation	E vs M	0.000623556	53
epithelial cell proliferation	M v RE	0.006007269	31
positive regulation of epithelial cell proliferation	E vs M	0.000169584	34
positive regulation of epithelial cell proliferation	M v RE	2.89E-06	37
positive regulation of epithelial cell proliferation	M v RE	0.018885374	17
regulation of mesenchymal cell proliferation	E vs RE	0.000172471	11
regulation of mesenchymal cell proliferation	M v RE	0.005028173	10
mesenchymal cell proliferation	E vs RE	0.000297495	12
mesenchymal cell proliferation	M v RE	0.000910719	13
positive regulation of fibroblast proliferation	E vs RE	0.007400475	11
positive regulation of fibroblast proliferation	E vs M	0.001181428	14
fibroblast proliferation	E vs M	0.010283961	16
Cellular Migration			144
tissue migration	E vs M	3.11E-14	61
tissue migration	E vs RE	3.49E-12	52
tissue migration	M v RE	2.41E-09	39
regulation of epithelial cell migration	E vs M	1.83E-11	48
regulation of epithelial cell migration	E vs RE	2.81E-09	40
regulation of epithelial cell migration	M v RE	7.38E-09	33
regulation of epithelial cell migration	M v RE	0.000244776	33
regulation of smooth muscle cell migration	E vs M	2.86E-07	23
regulation of smooth muscle cell migration	M v RE	0.000239404	14
regulation of smooth muscle cell migration	M v RE	0.003309968	16
epithelium migration	M v RE	2.35E-05	42
epithelial cell migration	M v RE	4.54E-05	41
Stemness			48
stem cell development	E vs M	0.001028317	16
stem cell proliferation	E vs M	0.017480962	18
stem cell proliferation	M v RE	0.044933608	12
stem cell division	E vs RE	0.027748376	8
positive regulation of stem cell differentiation	M v RE	0.03365355	6
regulation of stem cell proliferation	E vs M	0.035332065	12

Biological Functions/Pathways	*Comparison*	*padj range*	*# DE genes*
Metabolism			163
positive regulation of small molecule metabolic process	E vs RE	0.000348475	22
regulation of small molecule metabolic process	E vs M	0.000745334	42
regulation of small molecule metabolic process	M v RE	0.024347284	36
aldehyde dehydrogenase (NAD+) activity	M v RE	0.001808179	7
polysaccharide metabolic process	M v RE	0.007501153	12
polysaccharide metabolic process	E vs M	0.013601786	15
polysaccharide metabolic process	E vs RE	0.04046331	12
regulation of ATP metabolic process	E vs RE	0.009057084	13
phosphatidylinositol metabolic process	E vs RE	0.018908829	16
phosphatidylinositol metabolic process	E vs M	0.009811476	19
cellular polysaccharide metabolic process	M v RE	0.0111419	11
amino sugar metabolic process	E vs M	0.012888703	8
regulation of cellular ketone metabolic process	E vs M	0.016539009	18
regulation of cellular ketone metabolic process	M v RE	0.026416447	18
hexose metabolic process	M v RE	0.023065391	26
hexose metabolic process	E vs M	0.048632048	26
regulation of polysaccharide metabolic process	E vs M	0.026247261	8
NAD+ nucleosidase activity	E vs RE	0.032040672	4
cellular polysaccharide metabolic process	E vs M	0.032635529	13
NADPH oxidase complex	M v RE	0.032806102	3
oxidoreductase activity, acting on NAD(P)H, oxygen as acceptor	M v RE	0.039744915	4
Cancer			214
Proteoglycans in cancer	E vs M	1.82E-06	52
Proteoglycans in cancer	M v RE	1.27E-06	48
Proteoglycans in cancer	E vs RE	0.000204	35
Breast cancer	E vs M	0.005959	31
Breast cancer	M v RE	0.001296	30
Breast cancer	E vs RE	0.001748	25
Small cell lung cancer	E vs M	0.01103	21
Small cell lung cancer	M v RE	0.017449	18
Small cell lung cancer	E vs RE	0.023162	15
Prostate cancer	E vs M	0.011518	22
Prostate cancer	M v RE	0.017449	19
Prostate cancer	E vs RE	0.004617	18
Choline metabolism in cancer	E vs M	0.019341	21
Transcriptional misregulation in cancer	E vs M	0.034637	34
Transcriptional misregulation in cancer	M v RE	2.75E-05	42
Transcriptional misregulation in cancer	E vs RE	0.034835	25
Bladder cancer	M v RE	0.003617	12
Bladder cancer	E vs RE	0.049247	8
Gastric cancer	M v RE	0.010737	27
Gastric cancer	E vs RE	0.008585	23
Non-small cell lung cancer	M v RE	0.043233	14
Central carbon metabolism in cancer	E vs RE	0.005668	14
PD-L1 expression and PD-1 checkpoint pathway in cancer	E vs RE	0.017637	15
Colorectal cancer	E vs RE	0.037883	14
Pancreatic cancer	E vs RE	0.049247	12

Figure S5. Top significantly enriched biological functions or pathways derived from the 4211 differentially expressed genes across E, M and RE cells.

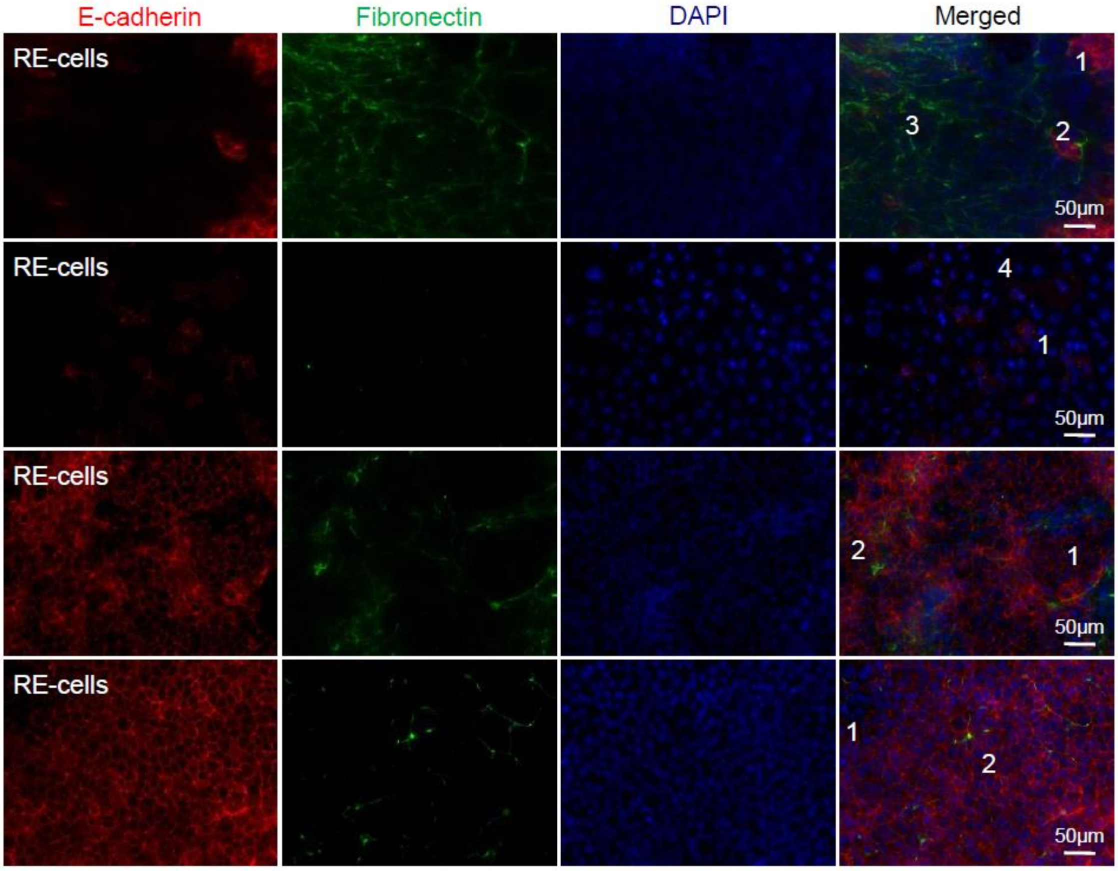

Figure S6: The 4 RE cell subpopulations co-exist spatially. (a) Representative images of different microscope fields of RE cells stained for E cadherin (red) and Fibronectin (green), displaying the 4 RE cells subpopulations labelled from 1–4: (1) E-cadherin^{+}/Fibronectin^{-}, (2) E-cadherin^{+}/Fibronectin^{+}, (3) E-cadherin^{-}/Fibronectin^{+} and (4) E-cadherin^{-}/Fibronectin^{-}. DAPI (blue) is also represented, and all channels are merged.

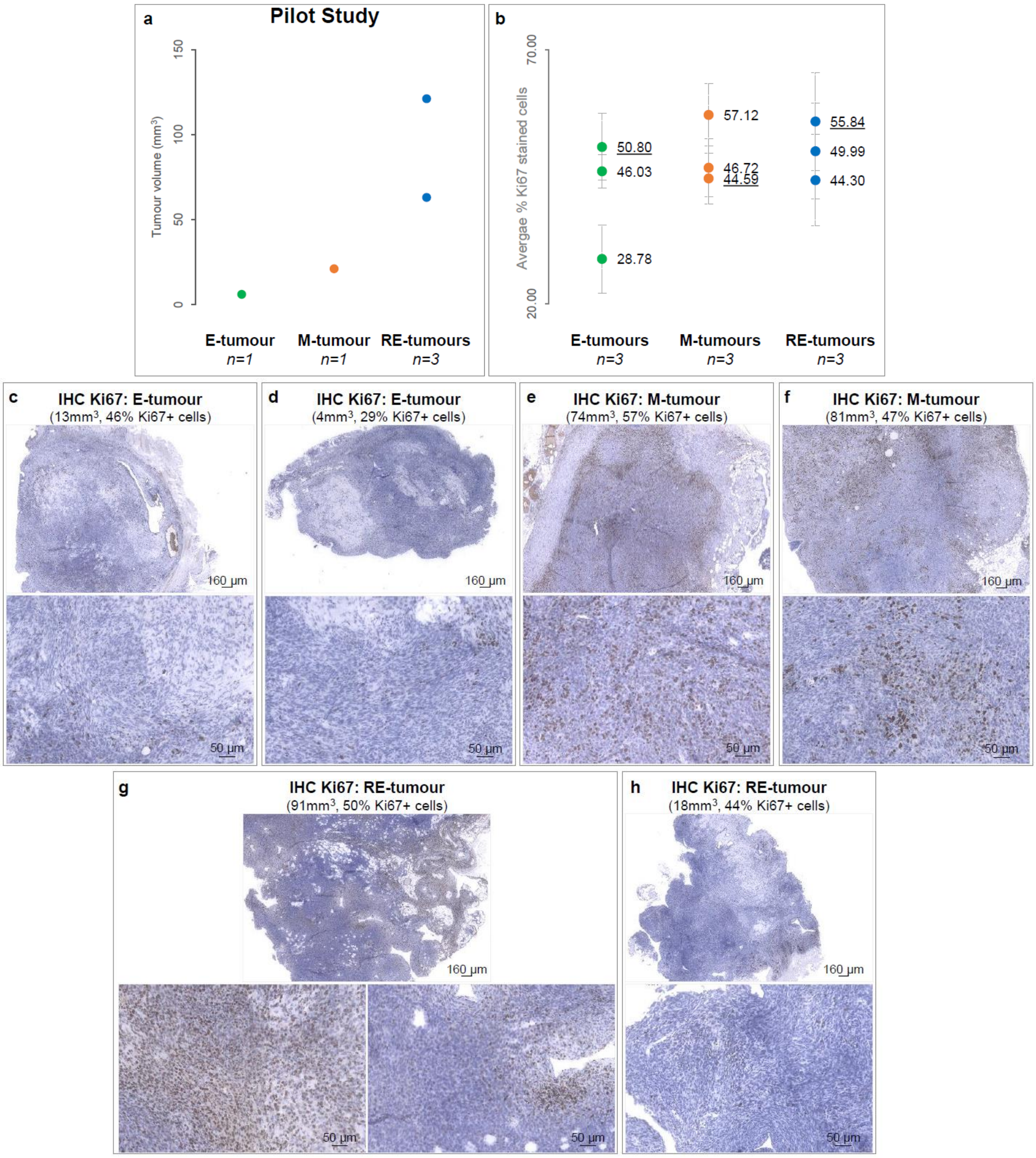

Figure S7. Mice experiments. (a) Pilot in vivo tumourigenicity assay for E, M and RE cells, with M and RE cell-originated tumours having larger volumes than those of E cells. (b) Average percentage of cells positive for Ki 67 staining in 3 E tumours, 3 M tumours and 3 RE tumours. (c–h) Representative images of immunohistochemistry staining for Ki 67 in 2 E tumours, 2 M tumours and 2 RE tumours. Top and bottom images show different magnifications.

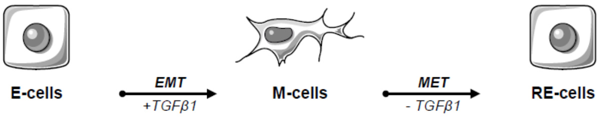

	E-cells	M-cells	RE-cells
**Brightfield morphology** *(Phenotype)*	Cobblestone	Fibroblastoid	Cobblestone
**Proliferation assay** *(BrdU average %)*	High (49%)	Low (34%)	High (52%)
**Wound healing assay** *(Wound closing pattern)*	Collective	Single-cell	Collective & Single-cell
**Focus formation assay** *(Phenotype)*	Dome-like structures	*Foci*	Dome-like & *Foci*
***In vivo* Tumorigenicity** *(Tumor volume)*	Low (3-25 mm^3)	High (32-343 mm^3)	High (5-304mm^3)
**First-Passage Mammosphere-forming eficiency** *(Average %)*	Low (0.5%)	High (1.2%)	High (1.2%)
**Metabolic signature** *(WB: HKII, LDH, ND1, NDUFS3, Rate of lactate produced per glucose consumed)*	OxPhos Active & Low Lactate	OxPhos Inactive & High Lactate	OxPhos Active & Low Lactate
**Phenotypic heterogeneity** *(IF:E-cadherin/Fibronectin)*	**Homogeneous** E-cadherin$^+$ / Fibronectin$^-$	**Homogeneous** E-cadherin$^+$ / Fibronectin$^+$ *(Non-functional E-cadherin)*	**Heterogeneous** E-cadherin$^+$ / Fibronectin$^-$ E-cadherin$^+$ / Fibronectin$^+$ E-cadherin$^-$ / Fibronectin$^+$ E-cadherin$^-$ / Fibronectin$^-$

Figure S8. Summary of the phenotypic and functional properties of E, M and RE cells. Properties analysed were brightfield morphology, proliferation, wound healing closure, first passage mammosphere-forming efficiency, focus formation ability, phenotypic heterogeneity by E-cadherin/Fibronectin immunofluorescence and in vivo tumourigenicity (final tumour volumes).
